# Using the Precision Lasso for gene selection in diffuse large B cell lymphoma cancer

**DOI:** 10.1186/s43046-023-00172-5

**Published:** 2023-06-26

**Authors:** Rashed Pourhamidi, Azam Moslemi

**Affiliations:** 1 Non Communicable Diseases Research Center, Bam University of Medical Sciences, Bam, Iran; 2 Department of Biostatistics, School of Medicine, Arak University of Medical Sciences, Sardasht, Basij Square, Arak, Markazi Province Iran

**Keywords:** Gene expression, High-dimensional, Precision Lasso, Lymphoma cancer

## Abstract

**Background:**

Gene selection from gene expression profiles is the appropriate tool for diagnosing and predicting cancers. The aim of this study is to perform a Precision Lasso regression model on gene expression of diffuse large B cell lymphoma patients and to find marker genes related to DLBCL.

**Methods:**

In the present case–control study, the dataset included 180 gene expressions from 14 healthy individuals and 17 DLBCL patients. The marker genes were selected by fitting Ridge, Lasso, Elastic Net, and Precision Lasso regression models.

**Results:**

Based on our findings, the Precision Lasso, the Ridge, the Elastic Net, and the Lasso models choose the most marker genes, respectively. In addition, the top 20 genes are based on models compared with the results of clinical studies. The Precision Lasso and the Ridge models selected the most common genes with the clinical results, respectively.

**Conclusions:**

The performance of the Precision Lasso model in selecting related genes could be considered more acceptable rather than other models.

## Introduction

Lymphomas are a group of malignant tumors that involve lymphocytic cells or the immune system. These diseases often originate in the lymph nodes but may be diagnosed first in extranodal tissues [1]. Lymphoma is divided into two types: Hodgkin’s and non-Hodgkin’s. Non-Hodgkin’s lymphoma (NHL) is a group of lymphoid-derived malignancies that are classified according to their clinical and biological characteristics. Non-Hodgkin’s cancer is one of the most common blood cancers. It is the eighth most common cancer in men and the eleventh most common cancer in women [2]. Non-Hodgkin’s lymphoma has several subgroups, including diffuse large B cell lymphoma (DLBCL), Burkitt lymphoma (BL), mantle cell lymphoma (MCL), gastric mucosa-associated lymphoid tissue (MALT), follicular lymphoma (FL), and others [3].

Diffuse large B cell lymphoma is the most common subtype of NHL lymphoma, accounting for 30% to 40% of all newly diagnosed cases [4]. NHL is the seventh most common cancer in the USA, with 19.6 new cases per 100,000 people between 2012 and 2016. The 5-year relative survival rate is 63% for DLBCL and 88% for FL. In recent years, many studies have confirmed that genetic factors are closely related to DLBCL [5, 6].

Microarray technology has advanced rapidly in biotechnology. In fact, molecular hybridization tests that rely on light visualization are now feasible in the area of nanotechnology in DNA microarrays. The two main uses of DNA chips are studies of transcriptomic and genetic mutations. In humans, the transcriptome is used to study differences in the genes expression levels in natural cells compared to tumor cells [7].

Advancements have been made in diagnostic and therapeutic technologies, but DLBCL is not yet predictable. Researches have shown that microarray technology has the potential to diagnose and predict cancer. In addition, the microarray expression profile can differentiate cancer based on cellular nature and growth stage. Therefore, microarray plays an important role in the discovery of cancer-related genomic abnormalities [3].

The technology for measuring gene expression levels and assessing variability for big data is a high-dimensional technology. Due to the large number of variables, it is not possible to use the classical hypothesis test. In other words, in the classical hypothesis tests, each variable tests independently. So, microarray data could be used for linear regression models, which simultaneously tests all variables. However, it is not possible to estimate the parameters with a linear regression model, and special methods should be used to reduce the number of variables or to ignore the minimizing the sum of squared errors [8].

In 1970, Harley and Kennard introduced Ridge regression model by adding the term “penalty” to the estimator of the ordinary least square. They tried to fix or reduce the sum of squared errors by using the penalty function on the parameters of the regression model. Therefore, the Ridge regression estimator in high-dimensional data was able to estimate the parameters using a linear combination of the estimator of the ordinary least square [9]. In 1996, Tibshirani introduced the Lasso regression model in which used the method of dimension reduction variables. He also used the method of minimizing the sum of square error to estimate the parameters. In this model, the number of parameters is controlled using a “penalty” function on the sum of the absolute values of the regression model coefficients. Despite solving the problem of estimating the parameters in multiple regression, the Lasso in the following two conditions does not provide a good result, which are:

1. If the two explanatory variables are highly correlated, they have a very similar effect on the response variable
2. If the explanatory variables are collinear

In the above conditions, the Lasso randomly selects one of the variables and causes the wrong result [8]. Zou and Hastie, in 2005, proposed the Elastic Net regression model.

The Elastic Net model combined the Lasso and Ridge with the placement of the second degree penalty equations. This model involved both the dimension reduction and the least squares estimation [10]. In the following years, many methods have been introduced to solve these two problems; a method that solves both of the above problems was proposed by Wang et al. in 2018 under the title of the Precision Lasso regression model [8].

The present study uses gene expression data from DLBCL patients that have been extracted by microarray technology. In this type of high-dimensional data, a high correlation between variables is also a problem. This study aims to apply Precision Lasso model on microarray data of DLBCL patients and finding gene markers related to DLBCL. Also, Precision Lasso compares with different penalty models. Therefore, patients benefit from more effective treatment opportunities by diagnosing and predicting the DLBCL cancer.

## Methods

**Fig. 1:**
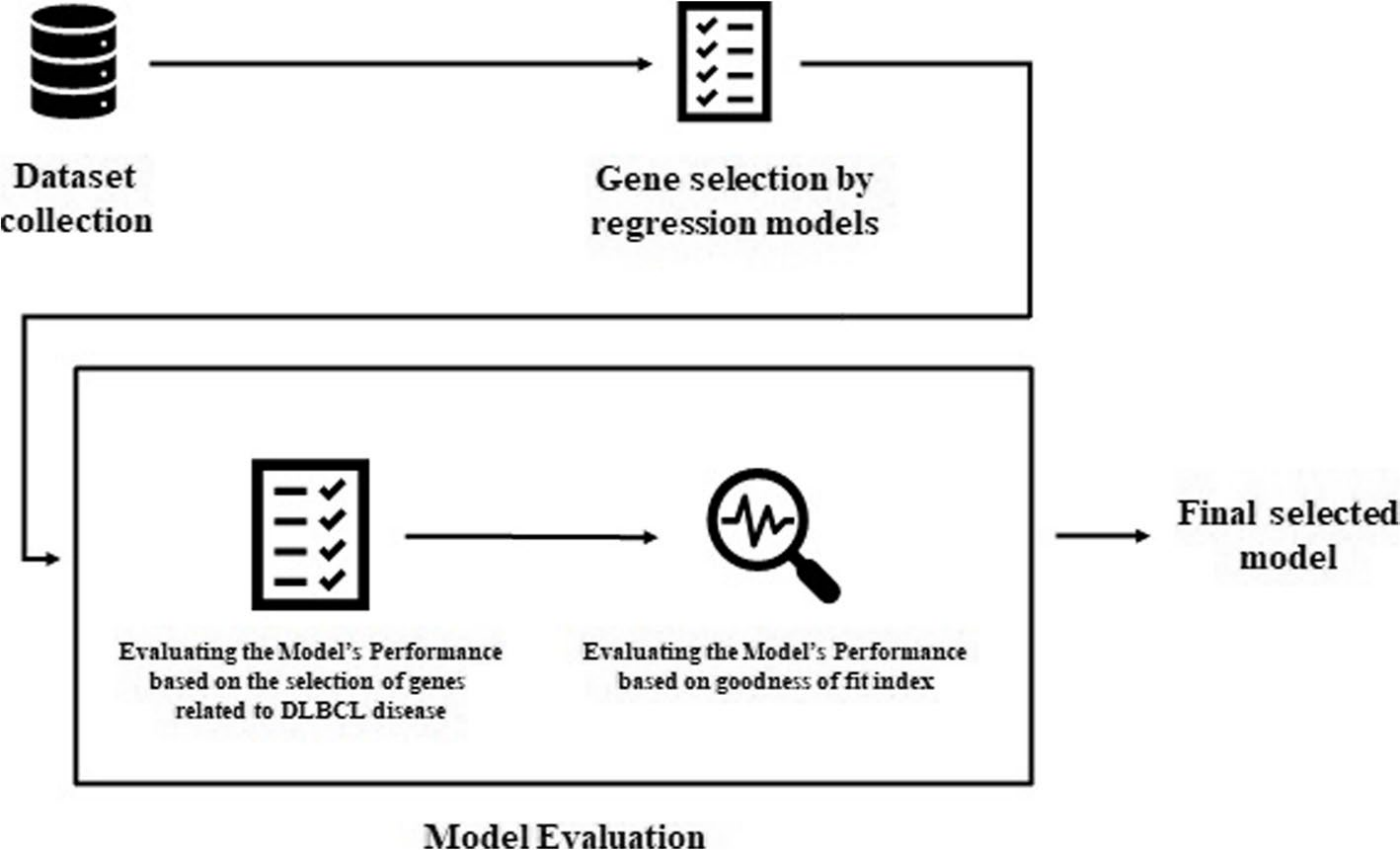
Steps of conducting the research

The methods used in this research are consistent with the related guidelines. The steps for conducting this research are presented in Fig. 1. Overall, the method includes dataset collection, gene selection by regression models, and model evaluation which is described in the following sections.

### Dataset collection

In the present case–control study, DLBCL data was used, which included 180 genes expression and 31 individuals. The data is available on the following site: https://www.ncbi.nlm.nih.gov/. The dataset includes blood samples from 31 donors, including 14 healthy individuals and 17 DLBCL patients. The notable point about the dataset is that when donating blood, people have no symptoms of the disease and are healthy enough to donate blood. According to Jorgensen et al., this is the first study of the microarray expression profile of apparently healthy individuals taken several years before the diagnosis of DLBCL [11].

### Gene selection

According to the dataset of the study, the most appropriate regression models were processed on these data. Regression models include the Ridge, the Lasso, the Elastic Net, and the Precision Lasso.

#### Shrinkage regression models

When the number of variables *p* is greater than the number of observations (*p* ≫ *n*), the ordinary least square method cannot be used to estimate linear regression coefficients. Another issue is determining the number of independent variables that should be used in the model. As the number of variables increases, over-fitting occurs, and as they decrease, we may encounter under-fitting.

To solve the problem of estimating parameters in high-dimensional data in the last two decades, many methods were proposed based on the dimension reduction and the converted minimum squared error estimator. Here, four different penalty methods are described with their advantages and disadvantages.

#### Ridge regression model

The best way to estimate the regression model parameters, due to the lowest error, is the ordinary least square method. However, it cannot be expected minimum variance for the estimators. Therefore, we need to find a way to select the right number of estimators. The application of Ridge regression is clarified in such situations. The estimator of Ridge regression is not unbiased but has a smaller variance than the ordinary least square method. In the ridge regression model, using the constraint ∥*β*∥^2^ ≤ *C* ^2^ on the parameters of the regression model, it tries to fix or reduce the sum of the squares of the parameters, so this constraint was added by the ordinary least square method.

One of the features of the Ridge regression model is that the penalty function reduces the coefficients to zero but does not make any of them zero. Of course, this does not apply to a so large *λ*. This feature challenges the interpretation of a model with a large number of variables [9].

#### Lasso regression model

The Lasso regression model provides a suitable method for modeling the response variable based on the lowest and most appropriate number of explanatory variables. This method separates the more suitable variables from the rest of the variables by providing a simpler model. That is why it is known as the Lasso method, which is a Canadian word meaning snare. In 1996, Robert Tibshirani, by using a penalty function on the sum of the absolute values of the regression model coefficients, controlled the number of parameters. In this condition, the sum of the squared estimate of errors of the Lasso model writes as follows:

1 $$\sum\nolimits_{i=1}^{N} \left( y_{i} - \beta_{0} - \sum\nolimits^{p}_{j=1} \beta_{j} x_{ij}\right)^{2} + \lambda \sum\nolimits_{j} \left| \beta_{j} \right|$$

*λ* is a regulating parameter, meaning that if its value is zero, the model will become linear regression, and all variables will be present in it. If its value increases, the number of explanatory variables in the model will decrease. One of the main goals of the Lasso is to improve the interpretation of the model by determining a smaller subset of explanatory variables that have the most effect [7].

#### Elastic Net regression model

The Elastic Net regression model was introduced by Zu and Hasti. Elastic means flexibility. In fact, the Elastic Net model is a combination of Lasso and Ridge models and uses second degree penalties. This method is used when the Lasso cannot select the grouping variable by one category and ignore the other categories. Using this model can be useful for the dataset with high correlation [10].

#### Precision Lasso regression model

The regular regression model, introduced by Wang et al. as Precision Lasso proved the instability and inconsistency in the Ridge, Lasso, and Elastic Net models primarily by using a condition called irrepresentable. The condition is as follows:

2 $$\left|{\left({x}^{\left(2\right)}\right)}^{T}{x}^{\left(1\right)}{\left({\left({x}^{\left(1\right)}\right)}^{T}{x}^{\left(1\right)}\right)}^{-1}sign\left({\beta }^{\left(1\right)}\right)\right|<1-\eta$$

In this condition, *x* ^(1)^ is a set of active variables *x* ^(2)^ is a set of inactive variables and η is a positive constant vector.

The instability of the Lasso points to its inability to detect the effects of correlated explanatory variables. Since correlated explanatory variables cannot analyze separately and by classical statistics, a simple way to achieve this goal is to determine similar weights for correlated variables. Considering the Trace Lasso regression model, a set of weights in which the correlated variables add to the other variables. Inconsistency is another disadvantage of the Lasso, which refers to the collinearity between variables. To solve the two problems of instability and inconsistency, for the first time, Wang et al. proposed *γ* a regulatory parameter to combine the two solutions. However, for example, if there is instability, *γ* = 1, and if there is inconsistency, *γ* = 0, and if there are both of them, *γ* = 1/2. The strategy introduced can be extended to other *ℓ* functions more simply. As an example, when the Response variable is dichotomous, by substituting *ℓ* with the negative in the likelihood logarithm, the Precision Lasso model is converted into a logistic regression model. This formula is applied in case–control data as those in the present study.

3 $$arg\ min\ell\left(x,\gamma ;\beta \right)+\lambda \Vert \left[{\gamma \left({x}^{T}x\right)}^\frac{1}{2}+\left(1-\gamma \right){\left({x}^{T}x+\mu I\right)}^{-\frac{1}{2}}\right]diag\left(\beta \right)\Vert$$

In the present study, due to the high correlation of genetic data, we tried to find cancer-related gene markers using the above four penalty methods [8].

#### Model evaluation

We evaluated shrinkage regression models using two steps. In the first step, according to previous studies, the expressed genes caused by DLBCL disease were identified. Then, we compared the genes that were selected using the models with the identified genes. In the next step, the holdout method was used with 10 folds. Then, the goodness of fit of regression models was compared based on the area under the ROC curve (AUC) and average precision score (AP-Score) [12].

Analysis of gene expression data was performed using R 3.6.2 and Python 2.7 software.

## Results

This study applied four penalty regression models, including the Ridge, the Lasso, the Elastic Net, and the Precision Lasso regression models, to select best genetic markers from the DLBCL cancer gene expression dataset. This dataset consists of 180 genes belonging to 31 individuals. These include 17 DLBCL patients and 14 healthy people. The dataset includes two challenges: the very high ratio of the number of variables to individuals and a high correlation between the genes. Therefore, selecting the more effective genes in the model would better predict DLBCL cancer. Four statistical models were fitted to the gene expression dataset. The maximum twenty genes with the highest coefficient in each regression models were selected and were compared with the DLBCL cancer-related genes based on results of clinical studies.

Table 1 showed the selected genes by regression models that had high level of expression related to DLBCL cancer based on clinical studies.

**Table 1:** Selected genes by regression models with high level of expression genes related to DLBCL cancer based on clinical studies

miRNA	Regression model			
	Lasso	Ridge	Elastic Net	Precision Lasso
hsa-let-7i-3p		✓		✓
hsa-let-7b-3p			✓	
hsa-miR-18a-3p		✓		✓
hsa-miR-20a-3p				✓
hsa-miR-27a-3p	✓		✓	
hsa-miR-29a-5p		✓		✓
hsa-miR-33a-5p		✓		
hsa-miR-103a-3p			✓	
hsa-miR-107			✓	
hsa-miR-126-3p			✓	
hsa-miR-197-3p	✓			
hsa-miR-200a-3p				✓
hsa-miR-296-5p		✓		
hsa-miR-326		✓	✓	✓
hsa-miR-331-3p		✓		✓
hsa-miR-421		✓		✓

Table 2 showed the selected genes by regression models that had low level of expression related to DLBCL cancer based on clinical studies.

**Table 2:** Selected genes by regression models with low level of expression genes related to DLBCL cancer based on clinical studies

**miRNA**	Regression model			
	**Lasso**	**Ridge**	**Elastic Net**	**Precision Lasso**
hsa-miR-10a-5p				✓
hsa-miR-30d-5p		✓		
hsa-miR-95				✓
hsa-miR-148a-3p			✓	
hsa-miR-154-5p		✓		✓
hsa-miR-190a				✓
hsa-miR-223-5p	✓	✓	✓	✓
hsa-miR-328		✓		✓
hsa-miR-342-3p	✓		✓	
hsa-miR-361-3p		✓		✓
hsa-miR-584-5p		✓		
hsa-miR-652-3p	✓		✓	

Based on results in Tables 1 and 2, the Precision Lasso had the biggest share in the selection of DLBCL cancer-related genes, followed by Ridge, Elastic Net, and Lasso.

**Fig. 2:**
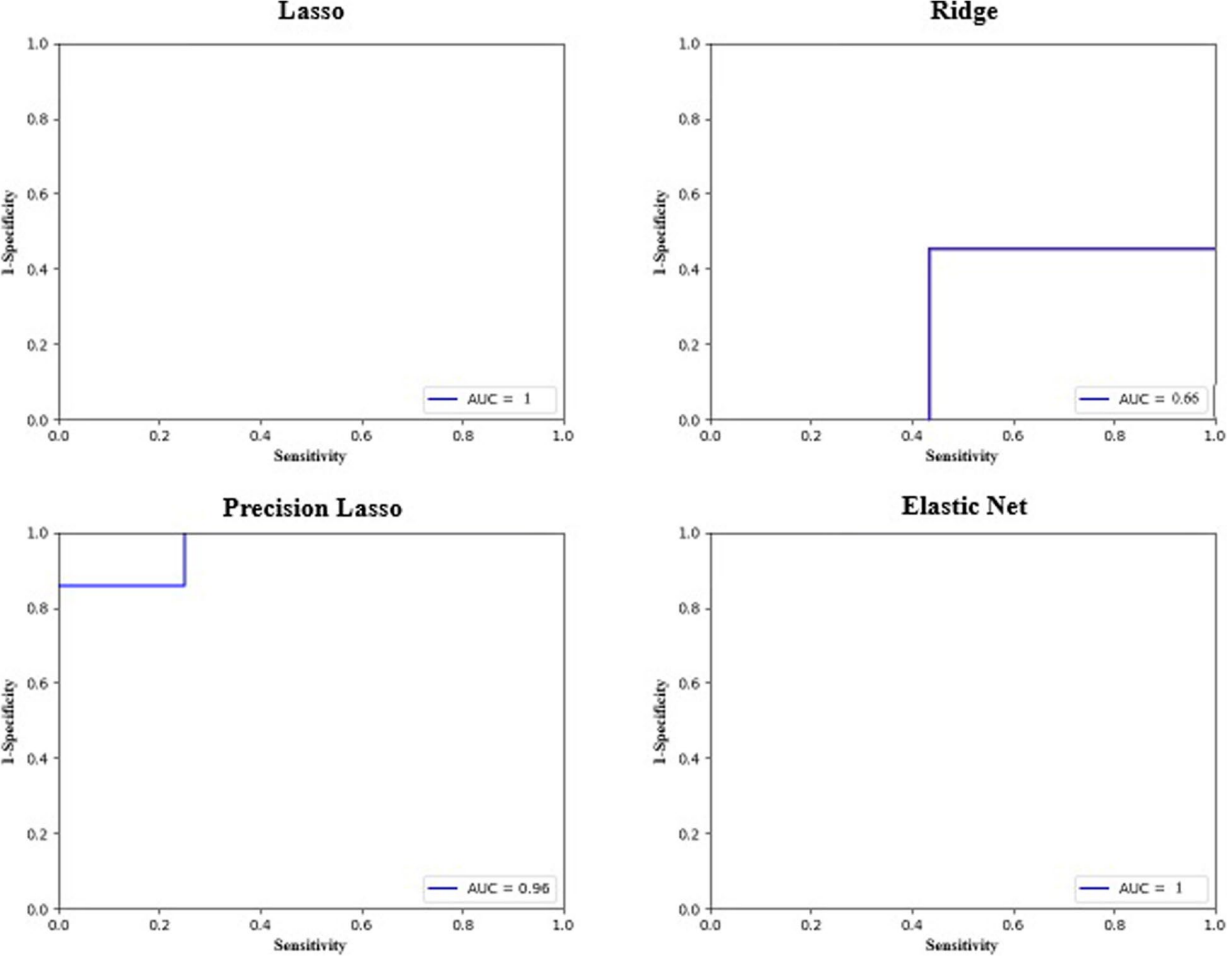
ROC curve for Ridge, Lasso, Elastic Net, and Precision Lasso models

Figure 2 showed ROC curves of binary logistic data for each models. The Ridge model had lowest AUC value and the Precision Lasso, Elastic Net, and Lasso had high AUC value.

Table 3 showed the goodness of fit index, AUC, and AP-Score for the understudy regression models based on holdout method. The Precision Lasso models had highest AP-Score. Also, the Lasso, Elastic Net, and Precision Lasso models had high AUC value.

**Table 3:** The goodness of fit test for regression models

Regression model	AUC	AP-Score
Ridge	0.66	0.45
Lasso	1	0.90
Elastic Net	1	0.90
Precision Lasso	0.96	0.98

Finally, the relationship of maximum the 20 genes that had the highest coefficient in the regression model in these four regression models were investigated with different types of cancer. According to Table 4, the Precision Lasso regression model selected the most DLBCL cancer-related genes.

**Table 4:** Relationship among the top 20 selected genes based on regression models and different types of cancer

**Method**	**miRNA**	**Disease**	**Target (DLBCL)**	**Reference**
**Ridge**	hsa-let-7i-3p	hepatocellular carcinoma (HCC)		[13]
	hsa-miR-361-3p	non-small cell lung cancer (NSCLC)		[14]
	hsa-miR-421	diffuse large B-cell lymphoma (DLBCL)	*	[15]
	hsa-miR-136-5p	carcinoma		[16]
	hsa-miR-223-5p	diffuse large B-cell lymphoma (DLBCL)	*	[17]
	hsa-miR-29a-5p	diffuse large B-cell lymphoma (DLBCL)	*	[18]
	hsa-miR-331-3p	diffuse large B-cell lymphoma (DLBCL)	*	[19]
	hsa-miR-425-3p	renal cell carcinoma (RCC)		[20]
	hsa-miR-296-5p	diffuse large B-cell lymphoma (DLBCL)	*	[21]
	hsa-miR-376a-3p	hepatocellular carcinoma (HCC)		[22]
	hsa-miR-335-5p	gastric cancer		[23]
	hsa-miR-584-5p	renal cell carcinoma (RCC)		[24]
	hsa-miR-500a-5p	breast cancer		[25]
	hsa-miR-33a-5p	lung cancer		[26]
	hsa-miR-18a-3p	diffuse large B-cell lymphoma (DLBCL)	*	[27]
	hsa-miR-328	diffuse large B-cell lymphoma (DLBCL)	*	[28]
	hsa-miR-154-5p	carcinoma		[29]
	hsa-miR-30d-5p	non-small cell lung cancer (NSCLC)		[30]
	hsa-miR-326	non-small cell lung cancer (NSCLC)		[31]
	hsa-miR-30e-3p	non-small cell lung cancer (NSCLC)		[32]
**Lasso**	hsa-miR-223-5p	diffuse large B-cell lymphoma (DLBCL)	*	[17]
	hsa-miR-197-3p	diffuse large B-cell lymphoma (DLBCL)	*	[33]
	hsa-miR-652-3p	non-small cell lung cancer (NSCLC)		[34]
	hsa-miR-27a-3p	diffuse large B-cell lymphoma (DLBCL)	*	[17]
	hsa-miR-342-3p	non-small cell lung cancer (NSCLC)		[35]
**Elastic Net**	hsa-miR-223-5p	diffuse large B-cell lymphoma (DLBCL)	*	[17]
	hsa-miR-197-3p	diffuse large B-cell lymphoma (DLBCL)	*	[33]
	hsa-miR-27a-3p	diffuse large B-cell lymphoma (DLBCL)	*	[17]
	hsa-miR-326	non-small cell lung cancer (NSCLC)		[31]
	hsa-miR-148a-3p	gastric cancer		[36]
	hsa-miR-652-3p	non-small cell lung cancer (NSCLC)		[34]
	hsa-miR-342-3p	non-small cell lung cancer (NSCLC)		[35]
**Precision Lasso**	hsa-miR-190a	diffuse large B-cell lymphoma (DLBCL)	*	[37]
	hsa-miR-208a	Non-small cell lung cancer (NSCLC)		[38]
	hsa-miR-10a-5p	renal cell carcinoma		[39]
	hsa-miR-182-5p	hepatocellular carcinoma (HCC)		[40]
	hsa-let-7i-3p	hepatocellular carcinoma (HCC)		[13]
	hsa-miR-20a-3p	diffuse large B-cell lymphoma (DLBCL)	*	[41]
	hsa-miR-1			
	hsa-miR-92b-3p	esophageal squamous cell carcinoma (ESCC)		[42]
	hsa-miR-29a-5p	diffuse large B-cell lymphoma (DLBCL)	*	[18]
	hsa-miR-361-3p	non-small cell lung cancer (NSCLC)		[14]
	hsa-miR-18a-3p	diffuse large B-cell lymphoma (DLBCL)	*	[27]
	hsa-miR-223-5p	diffuse large B-cell lymphoma (DLBCL)	*	[17]
	hsa-miR-95	Carcinoma		[43]
	hsa-miR-200a-3p	diffuse large B-cell lymphoma (DLBCL)	*	[44]
	hsa-miR-154-5p	Carcinoma		[29]
	hsa-miR-328	diffuse large B-cell lymphoma (DLBCL)	*	[28]
	hsa-miR-326	non-small cell lung cancer (NSCLC)		[31]
	hsa-miR-421	diffuse large B-cell lymphoma (DLBCL)	*	[15]
	hsa-miR-425-3p	renal cell carcinoma (RCC)		[20]
	hsa-miR-331-3p	diffuse large B-cell lymphoma (DLBCL)	*	[19]

## Discussion

The study used gene expression dataset from the DLBCL patients. Four penalty regression models were applied, including the Ridge, the Lasso, the Elastic Net, and the Precision Lasso.

In particular, these regression models are suitable for such dataset, including the number of explanatory variables greater than the number of observations, with a high correlation between variables. These models selected genes related to DLBCL cancer. The results were reported by statistical and clinical comparison. Among the regression models under study, Precision Lasso, Ridge, Elastic Net, and Lasso regression models selected genetic markers (high and low expression levels) associated with DLBCL cancer, respectively. Also, the top 20 genes were selected based on these regression models and compared with results of clinical studies. In this comparison, Precision Lasso regression and Ridge regression models were the most accurate, respectively, and Elastic Net and Lasso regression models selected the least number of genetic markers associated with DLBCL cancer.

In the following, the AUC and AP-Score were used to compare the goodness of fit of models. The ROC curve was plotted for the models. The Ridge model had the lowest area under ROC curve diagram, and the Precision Lasso, Elastic Net, and Lasso had highest AUC value. Also, the AP-Score was lowest for Ridge model, but the highest AP-Score was calculated for Precision Lasso. Based on the goodness of fit of the Precision Lasso, Lasso and Elastic Net models are very accurate.

The increasing importance of variable selection for high-dimensional data in various sciences has led to the introduction of new methods. Recently, the use of shrinkage methods has received much attention. In 2016, Padthe et al. showed that among the penalty regression models, the Elastic Net regression model performed better [45]. In 2018, Farhadi et al. compared the three models of Ridge, Lasso, and Elastic Net regression on simulated data. In this study, the Ridge regression model had the worst performance, and the Elastic Net regression model had the best performance [46]. In 2018, Wang et al. by comparison between different regression models on breast cancer gene expression showed that the Precision Lasso and Trace Lasso regression models were more accurate than other penalty regression models.

## Conclusion

According to our results, the performance of Precision Lasso regression model in selecting gene markers is more acceptable than other models. It suggests other regression models, including the Adaptive Lasso and Trace Lasso regression model use in future studies. There are also many data mining methods, such as machine learning, to compare with regression models. High-dimensional data in various sciences has expanded so much that a science called data science has been developed as an interdisciplinary science. This study was performed on a DLBCL dataset that had been extracted in a very small sample size with microarray technology. Also, it efforts theses regression models compare based on results of larger sample of microarray data.

## Data Availability

Datasets analyzed during the current study are available in the https://www.ncbi.nlm.nih.gov/geo/query/acc.cgi?acc=GSE117063 [11].

## Abbreviations

NHL: Non-Hodgkin’s lymphoma
DLBCL: Diffuse large B cell lymphoma
BL: Burkitt lymphoma
MCL: Mantle cell lymphoma
MALT: Gastric mucosa-associated lymphoid tissue
FL: Follicular lymphoma
AUC: Area under the ROC curve
AP-Score: Average precision score
